# Causal connectivity from right DLPFC to IPL in schizophrenia patients: a pilot study

**DOI:** 10.1038/s41537-022-00216-0

**Published:** 2022-03-07

**Authors:** Branislava Ćurčić-Blake, Claire Kos, André Aleman

**Affiliations:** 1 University of Groningen, Cognitive Neuroscience Center, Department of Biomedical Science of Cells and Systems, University Medical Center Groningen, Groningen, The Netherlands; 2 Shenzhen Key Laboratory of Affective and Social Neuroscience, Center for Brain Disorders and Cognitive Sciences, Shenzhen University, Shenzhen, China

**Keywords:** Schizophrenia, Neural circuits

## Abstract

Abnormal function and connectivity of the fronto-parietal network (FPN) have been documented in patients with schizophrenia, but studies are correlational. We applied repetitive transcranial magnetic stimulation (rTMS) to the dorso-lateral prefrontal cortex (DLPFC) and observed causal connectivity to the inferior parietal lobe (IPL). We hypothesized that patients with schizophrenia would have lower activation and slower reaction in the IPL following DLPFC stimulation. Thirteen patients with schizophrenia (SZ) and fourteen healthy controls subjects (HC) underwent rTMS at 10 Hz to the right DLPFC. Simultaneously, we measured brain activation in the IPL, represented as oxygenized hemoglobin (HbO) levels, using functional near-infrared spectroscopy (fNIRS). rTMS consisted of 20 trains of impulses at 10 Hz for 3 seconds, and 60 seconds waiting time. Using NIRSLab software, GLM was applied to estimate both hemodynamic response function (HRF) and its derivative. Following TMS to the DLPFC, SZ showed a smaller decrease in HbO levels in the bilateral IPL than HC (*p* = 0.05). Timecourse analysis revealed an immediate decrease in parietal HbO levels in HC, but not in SZ. This difference was significant (at a threshold level of *p* ≤ 0.05, with Bonferroni correction) for several time segments and channels in both rights and left IPL. Our findings suggest abnormal fronto-temporal connectivity in patients with schizophrenia, beyond a mere decrease or slowing of information processing. This is in line with the hypothesis of reduced fronto-parietal inhibition in schizophrenia.

## Introduction

Schizophrenia is a severe mental illness characterized by symptom dimensions such as positive and negative symptoms, cognitive deficits, and lack of insight into illness, amongst others^1–3^. At a neural level, schizophrenia is characterized by several brain abnormalities including dysconnectivity between brain areas^4,5^. Here, dysconnectivity refers to the wide range of abnormalities in both functional and anatomical brain connectivity. However, it is important to note that the abnormalities in the anatomical connectivity are not necessarily coupled linearly with abnormalities in functional connectivity^6,7^.

One of the most prominent deficits in schizophrenia is impairment in executive function. Executive function refers to higher-order cognitive processes necessary for daily functioning. The function includes sustained attention, i.e., vigilance, working memory, initiation, inhibition, set-shifting, and planning^8,9^. The neural correlates of the executive function are multifarious but consistently involve the fronto-parietal network (FPN) including the dorso-lateral prefrontal cortex (DLPFC) and parietal cortex^10^.

This impairment in executive function in patients with schizophrenia is associated with aberrant function of the dorso-lateral prefrontal cortex (DLPFC)^11^, parietal cortex^10^, and the fronto-parietal network (FPN)^12,13^. The FPN, also called central executive network (CEN), is involved in cognitive control and central executive function^14,15^. Differences between healthy control subjects and patients with schizophrenia in the FPN have been observed in relation to cognitive deficits during resting state^13,16^, during specific functions such as working memory^17–20^, and also at the anatomical level^21^. However, fMRI-based techniques cannot determine causal relationships of interactions between brain regions^22^ because regular neuroimaging methods are correlative rather than causal in nature. In order to infer causality from neuroimaging data, a direct external manipulation of neural activity is required.

In this study we investigated the causal relationship between the DLPFC and IPL by combining repetitive transcranial magnetic stimulation (rTMS) with functional near-infrared spectroscopy (fNIRS). rTMS directly affects the stimulated brain region and connected brain areas^23^ and therefore can be used to investigate causal interactions between brain regions. fNIRS is a method to measure levels of oxygenized and deoxygenized hemoglobin (HbO and Hb) using light in the near-infrared range^24,25^. Such measurements are not affected by magnetic fields (as in the case of fMRI and EEG). rTMS stimulation to the DLPFC at 10 Hz, which is globally considered excitatory^26^, may cause either a putative increase or decrease of activation in the IPL, depending on whether connections are inhibitory or excitatory^27^.

In healthy people, rTMS at 10 Hz delivered to the left DLPFC was shown to alter cognitive control and ERP^28^ and to alter resting-state functional connectivity of a network that involved anterior cingulate cortex, IPL, inferior frontal cortex, and posterior temporal cortex^29^. 10 Hz rTMS stimulation to either left or right DLPFC improved reaction time and neuronal efficiency during working memory performance in healthy adult participants^30^. Yamanaka and colleagues investigated the effects of rTMS at 5 Hz delivered to either right or left IPL in healthy adults^31^. They found that stimulation to the right but not to the left IPL improved functioning during a spatial working memory task and affected HbO levels in the frontal cortex. This suggests that the effects of high-frequency rTMS on specific nodes of the fronto-parietal network affect other nodes of the network. Based on such evidence, we expect that 10 Hz rTMS to the right DLPFC will affect HbO levels in bilateral IPL in healthy volunteers.

Further, given that the anatomical fronto-parietal connectivity is deficient in schizophrenia^21,32,33^, we expect the deficiency will reflect on the speed of the connection from one region to another. Thus, we hypothesized that patients with schizophrenia would have a slower change of HbO levels in IPL as a consequence of DLPFC stimulation, which will be reflected in lower strength of the derivative of estimated haemodynamic response function (HRF) and insignificant difference in the first 30 seconds of the HbO timecourse.

## Results

### Demographics

There was no significant difference in age and gender between the groups Table 1. The two groups differed in education (t(2,25) = 2.58, *p* = 0.016). All patients used antipsychotics, listed in Table 1. As expected, there was a difference in psychophatology scores between HC and Schizophrenia patients (t(2,24) = −10.8, *p* < 0.001). PANSS measures were not collected for HC. In patient group all PANSS values were >0.

**Table 1:** Demographic data of all participants.

	Healthy controls	Schizophrenia patients	HC vs Sczhi
	(*n* = 14)	(*n* = 13)	*p*-value
Age in years	36.3 (13.7)	39.2 (11.4)	0.1
Gender males/female	8/6	10/3	0.27
Education	6.3 (0.6)	5.5 (0.9)	0.016
Weight	75.9 (11.3)	95.2 (14.3)	0.001
Hight	177 (7)	185.5 (10.8)	0.02
AES_Total	24.9 (4.5)	47.5 (6.1)	<0.001
PANSS pos.	–	14.5 (5.7)	
PANSS neg.	–	17.1 (5.2)	
PANSS gen.	–	34.6 (8.8)	
PANSS tot.	–	66.1 (16.7)	
Duration of illness in years	–	7.0 (3.8)	
Medication [mg] Sum D2 receptor equivalent	–	66 (16)	
**List of antipsychotics**		**Number of patients**	
Clozapine		3	
Olanzapine		3	
Risperidone		2	
Aripirpazol		4	
Quetiapine		1	

The left column lists the demographic variables. The second and third columns from the left show average values of the variables across the group, with their standard deviations in brackets. Education level was rated according to a six point scale defined by Verhage, which ranges from primary school (1) to university level (6). Non-parametric test was used to test the group difference for gender (Chi-square).

### GLM

**Fig. 1:**
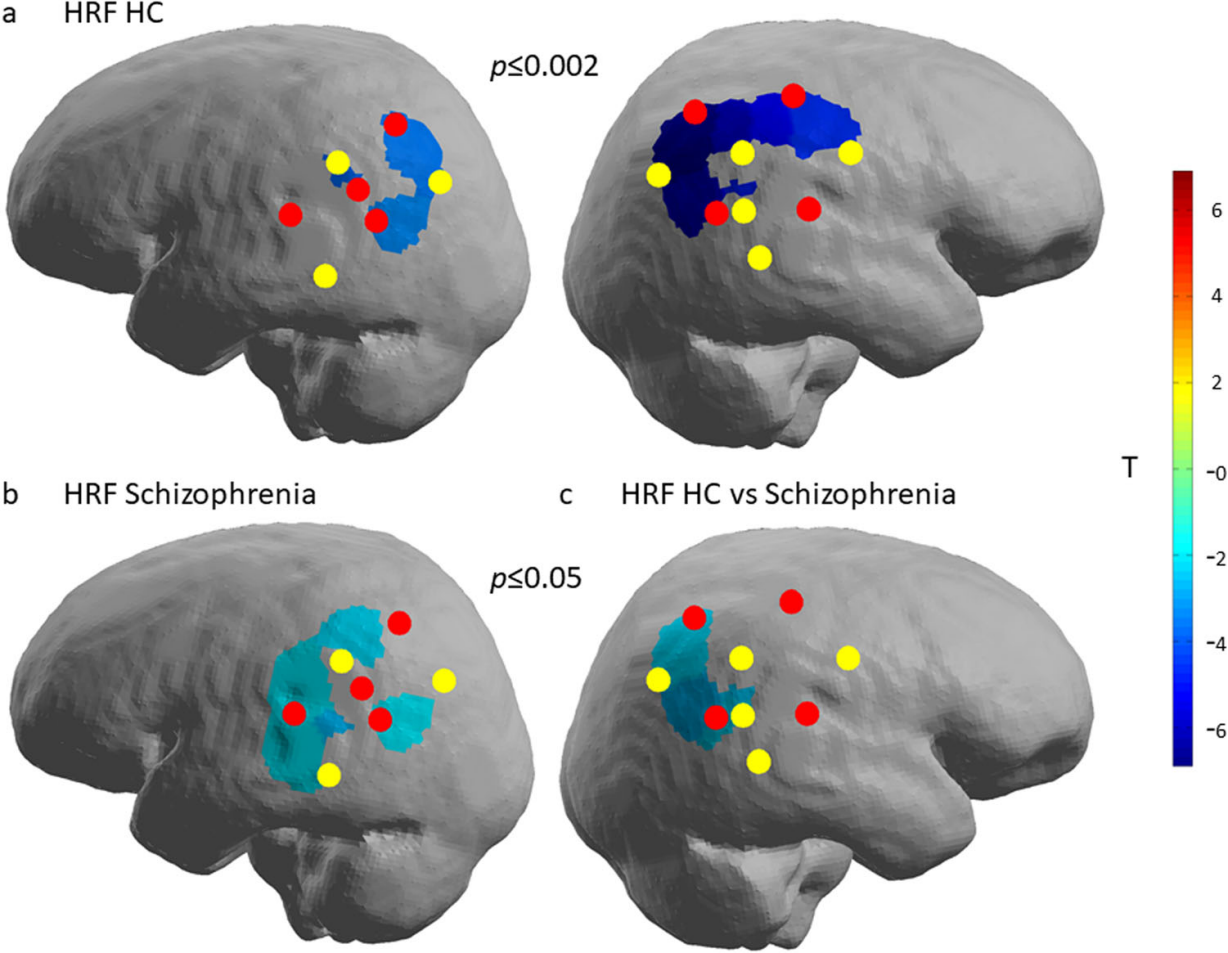
GLM results of group activation measured by HbO changes in ipsilateral and contralateral IPL as a consequence of 10 Hz rTMS delivered to the DLPFC. The red dots (sources) and yellow dots (detectors), same as in **a**, illustrate coverage by fNIRS. **a** GLM of HRF in HC threshold set at *p*_bonferroni_ ≤ 0.05 (or *p* ≤ 0.002). There was a significant decrease of activation in both hemispheres. **b** GLM of HRF in schizophrenia patients. The decrease of activation is significant at the threshold of *p*_uncorrected_ ≤ 0.05 only in the left hemisphere. There was no significant change of activation in the right hemisphere. **c** GLM of HRF of HC vs schizophrenia patients. The difference in activation was significant at threshold of *p*_uncorrected_ ≤ 0.05. After correction for multiple comparisons only significant in the right hemisphere. There was no significant difference in activation in the left hemisphere.

For GLM, HRF function was defined from 0 to 32s after the onset of the rTMS train. GLM revealed decreased levels of HbO in HC in bilateral IPL following the rTMS to the DLPFC (*p*_bonferroni_ = 0.05; Fig. 1a). For the schizophrenia group, decreased levels of HbO in left IPL following the rTMS to the DLPFC were observed (*p*_uncorrected_ = 0.05; Fig. 1b). This decrease was not statistically significant after correction for multiple comparisons. Patients differed from HC in HbO levels after rTMS, namely patients had higher levels of HbO compared to HC in the right hemisphere (at a significance level of *p* = 0.05, but not after correction for multiple comparisons; Fig. 1b). No difference in derivative of HRF was observed.

### Timecourses

**Fig. 2:**
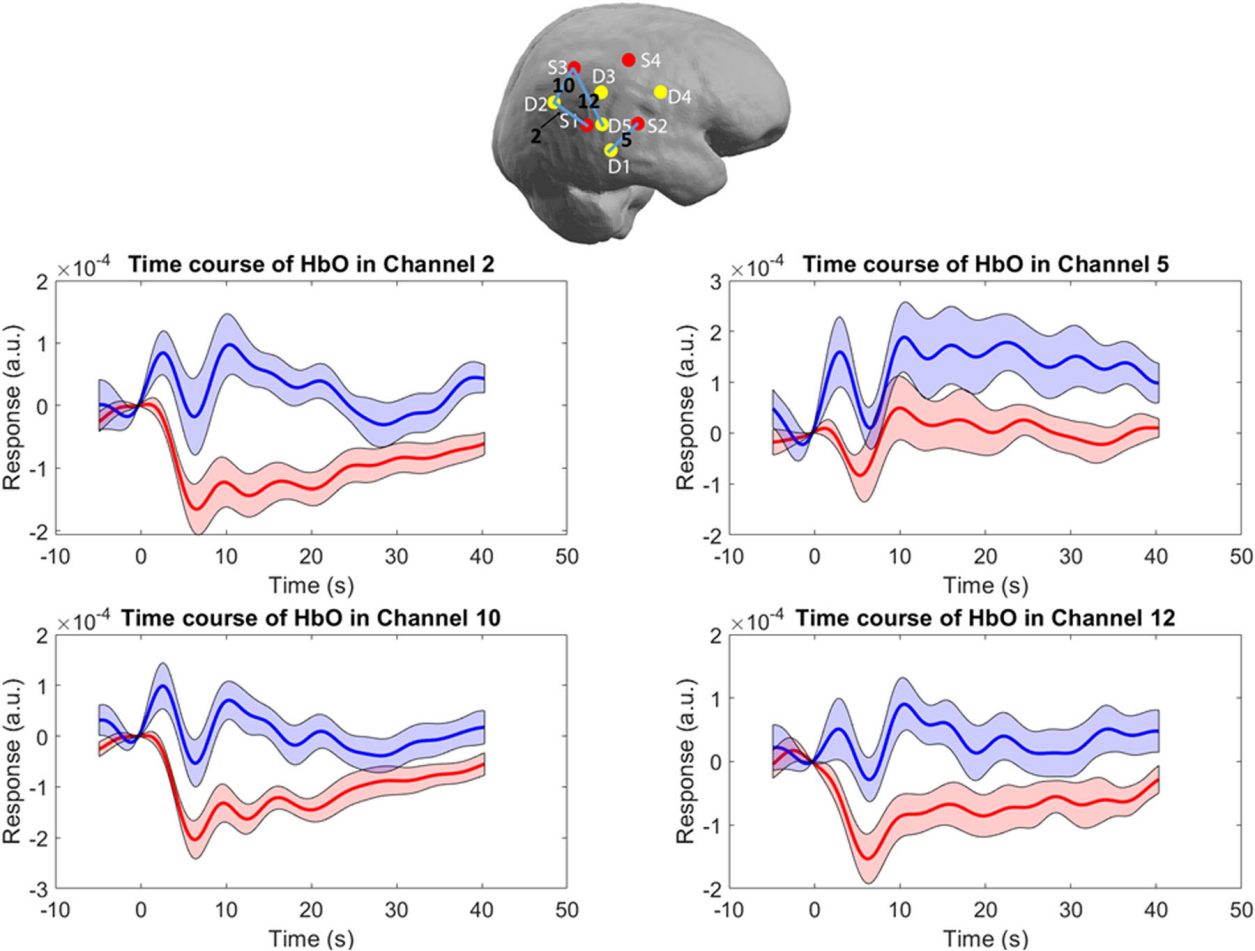
Timecourses of the HbO levels across the ipsilateral IPL after the 10 Hz rTMS delivered to the DLPFC. Top incision: Right brain hemisphere with distributed optodes (red and yellow dots are sources and detectors, respectively) and the channels depicted in the panels below. Black numbers correspond to the channel numbers, blue lines illustrate the path over which the channel data was collected. Panels middle and below: Solid lines represent grand averages of time courses and shaded areas represent standard error of mean (S.E.M.): red—healthy controls, blue—patients with schizophrenia. Here we depicted representative channels from ipsilateral hemisphere: Ch 2 posterior part of IPL, Ch 5—inferior part of IPL, Ch 10—superior part of IPL and 12 central part of IPL.

**Fig. 3:**
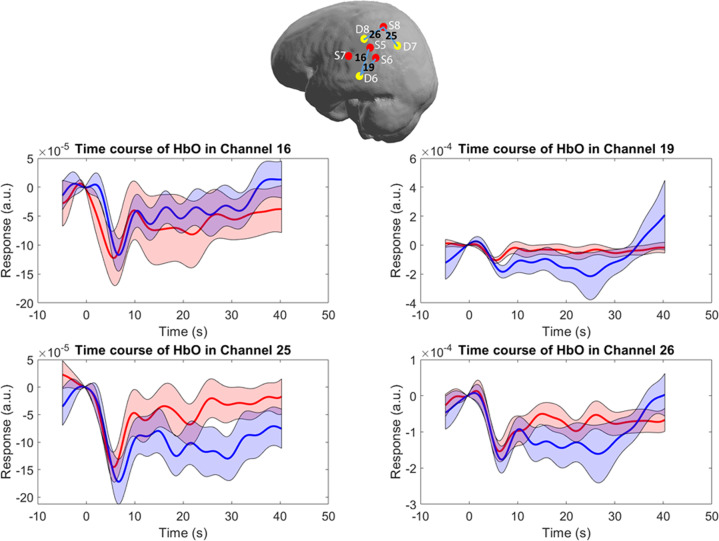
Timecourses of the HbO levels across the contralateral IPL after the 10 Hz rTMS delivered to the DLPFC. Top incision: Left brain hemisphere with distributed optodes (red and yellow dots are sources and detectors, respectively) and the channels depicted in the panels below. Black numbers correspond to the channel numbers, blue lines illustrate the path over which the channel data were collected. Panels middle and below: Solid lines are grand average and shaded areas present standard error of mean (SEM)): red—healthy controls, blue—patients with schizophrenia. Here we depicted representative channels from contralateral hemisphere: Ch 16—central part of IPL, Ch 19—inferior part of IPL, Ch 25—posterior part of IPL and 26 superior part of IPL.

In addition, there was a significant difference in timecourses between patients and HC following rTMS stimulation. Namely, while there was an immediate decrease in parietal HbO levels in HC, in SZ first an increase, followed by a decrease was observed (Figs. 2 and 3). This was significant (at a threshold level of *p*_Bonferroni_ = 0.05) for several time segments and channels in both right and left IPL (Table 2). There was no significant difference in the timecourses between HC and patients in the first time segment (0–5.1 s). The most significant difference was found in the ipsilateral IPL (the right IPL) in channels 2 (posterior), 10 (the most posterior), and 11–13 (superior channels), from 5 to 25 s. On the contralateral side, the significant difference occurred in two-time segments at two channels (25 and 26, the most superior and the most posterior channel).

**Table 2:** FDR corrected *p*-values of differences in the changes in HbO between healthy controls and patients with schizophrenia.

Channel number	IPL channel location	0–5.1 s	5.1–10.3 s	10.3–15.4 s	15.4–20.5 s	20.5–25.6 s
2	Right posterior IPL inferior part	10.869	0.210	**0.040**	**0.018**	**0.002**
10	Right posterior IPL	9.678	**0.003**	**0.002**	**0.000**	**0.000**
11	Right posterior IPL superior part	7.337	**0.036**	**0.021**	**0.009**	**0.027**
12	Right posterior IPL middle part	15.060	0.253	**0.067**	0.483	0.994
13	Right anterior IPL superior part	21.766	3.879	**0.010**	**0.014**	**0.034**
25	Left posterior IPL	10.715	**0.005**	0.293	4.872	7.550
26	Left posterior IPL superior part	18.468	6.601	**0.041**	0.919	2.208

The top column lists the time range (in seconds) for which the difference is calculated. Left column lists the channel numbers while second left lists the corresponding regions of IPL. Change is calculated compared to a baseline. *p*-values are corrected for multiple comparisons using FDR correction. Here, in bold we depicted channels with significant difference after correction for multiple comparisons.

## Discussion

In this study we examined causal functional brain connectivity from the right DLPFC towards bilateral IPL in patients with schizophrenia and healthy controls. Causal perturbations to the DLPFC were delivered by trains of rTMS while the instigated brain activation of the IPL was measured using fNIRS. We observed differences in activation of bilateral IPL as a consequence of DLPFC stimulation in patients with schizophrenia as compared to the healthy control group. Namely, patients with schizophrenia had an initial increase in HbO levels followed by a decrease on some channels. This was in contrast with the HbO levels in HC, which showed an immediate decrease in HbO levels following the 3 s stimulation, which was maintained over a period of at least 30 seconds. In addition, the subsequent decrease in HbO levels at the later time point after the onset of the stimulus in schizophrenia patients was less intensive than the decrease in HC along the whole timecourse of one event. This finding might indicate different information processing from the frontal to parietal lobe in schizophrenia patients as compared to control participants. Our finding is in line with the idea of impaired fronto-parietal inhibition in schizophrenia, which has been proposed to underlie impairments in schizophrenia such as lack of cognitive control^34–36^.

Dorso-lateral Prefrontal Cortex (DLPFC) is involved in a variety of cognitive functions important for healthy perception and daily functioning. Among others, DPLFC plays a crucial role in working memory^37^ and inhibition control as demonstrated using the Go/NoGo task^38,39^. Abnormalities in DLPFC connectivity with parietal areas have been repeatedly reported for patients with schizophrenia^40^.

The DLPFC and IPL are connected directly, via superior longitudinal fasciculus^41,42^ most probably the second branch of it (SLF II). They are a major part of fronto-parietal executive network^43,44^. It is difficult to discern whether the anatomical connections from the DLPFC to IPL are inhibitory or excitatory because of the limitations of the measurement methods for humans. Namely, the non-invasive MRI-based DTI methods, used for example by Thiebaut de Schotten and colleagues^41^, can only follow thick fibers, without information about directionality (i.e., afferent or efferent). Anterograde and retrograde tracer studies are invasive and only possible in primates such as monkeys but specifically for the DLPFC connections to IPL they do not match between humans and monkeys^42^. Another possibility to investigate inhibition, excitation, and directional connectivity in humans is by using TMS. Rogasch and colleagues^45^ reviewed the studies of cortical inhibition, excitation, and connectivity using TMS in schizophrenia. The studies that were summarized used paired TMS pulses to produce short-interval intracortical inhibition (SICI) or long-interval intracortical inhibition (LICI). Both SICI and LICI represent the relative amplitude reduction of motor evoked potentials (MEPs) by subthreshold conditioning stimuli events where conditioning stimulus is presented either short time (for SICI) or long time (for LICI) before the test stimulus. They explained their findings in terms of levels of neurometabolites such as γ-amino butyric acid (GABA). Deficits in GABAergic activity have previously been reported in schizophrenia^46^. Rogasch and colleagues^45^ found GABA_A_ receptor-mediated cortical inhibitory deficits in schizophrenia as well as in people with high risk and first-episode patients. These inhibitory deficits were both found on a local level (in PFC) and related to some long-range connections (including cortical, subcortical, and cerebellar connectivity). They suggested that the observed inhibitory deficits may lead to hyperexcitability in glutamatergic pathways and other network abnormalities in specific local neuronal populations that interact with long-range connections underlie cortical dysconnectivity in patients with schizophrenia. Unfortunately, none of the studies summarized were specific to DLPFC- IPL connection but rather related to motor cortices (see for example^47^). Nevertheless, they found the cortical inhibitory deficits in PFC in patients, which coupled with our findings and may suggest deficits in inhibition from DLPFC to IPL. In a similar fashion, Ferrarelli and colleagues^48^ used 0.4–0.6 Hz TMS stimuli delivered to several brain regions including the DLPFC and IPL while simultaneously measuring EEG in 20 healthy controls and 20 patients with schizophrenia. In addition to observing general slower prefrontal natural frequency of individuals with schizophrenia compared to healthy controls, they also found a reduction in TMS-related amplitude (ERSP) and synchronization (ITC) of beta/gamma-band EEG oscillations recorded at frontal/prefrontal sites in patients with schizophrenia compared with healthy control subjects. This was not observed for TMS delivered to parietal regions. The authors suggested that their findings argue against differences in neuronal excitability and likely reflect impairments in local cortical and thalamocortical circuits in schizophrenia. They also propose that a possible mechanism for the slowing of the prefrontal natural frequency might be due to deficits in GABAergic inhibition.

Given the disrupted fronto-parietal network in schizophrenia we expected to observe slower connectivity from frontal to parietal regions. We expected this to be reflected in the lower first derivative of the hemodynamic response. However, there was no difference for the first derivative. Surprisingly, the response was different in shape between groups. While for some channels a slower response was observed, for most of the channels we observed a different shape of the response. This suggests the intrinsically different fronto-parietal information processing in patients with schizophrenia as compared to healthy individuals.

We observed decreased HbO levels in parietal cortices as a consequence of stimulation in the healthy control group. The decreases in HbO levels suggest that there is an inhibitory stimuli arriving from DLPFC^49,50^. However, the relationship between the increase and decrease in HbO level is not linear with the activation of underlying neuronal tissue. The levels of HbO are rather associated with the cerebral metabolic rate of oxygen (CMRO_2_) and the cerebral blood flow (CBF) and the state of various blood vessels^50^. The prolonged decrease in HbO was observed previously after electrical stimulation^51^, intermittent photic stimulation^52^, relaxing 2-dimensional images presentation^53^, reading and picture observation task^54^ or it can vary per subject performing the same task (reading)^55^. Clearly, more studies are needed, using fNIRS and possibly rTMS combined with fMRI, to further elucidate this finding.

There are several limitations of this study that should be mentioned here. First, this was a pilot study, and therefore the sample size is relatively limited. Nevertheless, our sample was large enough to detect substantial differences between the groups. Regardless, follow-up studies are needed to confirm and expand on these results. Second, due to the thickness of the optodes, we could not measure the brain activation at the stimulation site because the coil would be too far from the scalp. As a consequence, we could not directly establish increased or decreased activation in the DLPFC. In general, this type of stimulation delivered to the motor cortex causes increased motor cortex excitability, reflected in increased activation (reviewed in ref. ^26^). However, this is dependent on the intensity of the stimulation. Thus, we suggest that the excitation of the DLPFC caused a decreased activation in the IPL in our measurements, but we cannot confirm the former with our equipment. In addition, in this pilot study we did not use sham condition, nor was this study placebo-controlled. It may be possible that rTMS could cause flinching or tensing of facial muscles which in turn could cause movement artefacts. While these side-effects occurred in some patients during the subsequent iTBS treatment, we neither observe either flinching nor tensing during the rTMS stimulation reported here. Finally, all patients were using antipsychotics at the time of the study. The calculated dopamine equivalent dose^56^ was relatively homogeneous (66 ± 16)mg per day. Therefore, while antipsychotic medication influences the brain activation in several brain regions, although not consistently in the DLPFC^57,58^, our study is not suitable to investigate the effects of medication. Future studies could include patients that never received medication or are not using it at the time.

In conclusion, we investigated fronto-temporal connectivity using near-infrared spectroscopy combined with rTMS. In this way we could investigate effective connectivity, meaning an actual effect that the DLPFC exerts on the IPL in healthy controls and in patients with schizophrenia. We found that 10 Hz rTMS delivered to the right DLPFC induces decreased activation of the right and left IPL. In schizophrenia patients, however, this effect was opposite and in the same channels the stimulation produced increased activation. This suggests that there may be aberrant fronto-temporal connectivity in patients with schizophrenia, beyond mere decrease or slowing of information processing. Future brain stimulation studies should further investigate this observed opposite effect of rTMS on distal brain regions and possible implications for rTMS treatment strategies.

## Methods

### Participants

Thirteen patients and fourteen healthy controls (HC) underwent repetitive transcranial magnetic stimulation (rTMS) to the right DLPFC. Patients also participated in a larger study into the treatment of apathy with intermittent theta-burst stimulation (iTBS) in schizophrenia after completing this pilot. Schizophrenia diagnosis was established by a clinician according to DSM V criteria. The diagnostic category was subsequently confirmed using the MINI plus interview^59^. The severity of symptoms was assessed using the positive and negative syndrome scale (PANSS) and the Apathy Evaluation Scale (AES). Exclusion criteria were comorbidities of severe mood or neurological disorders (including epilepsy or family history of epilepsy), brain surgery, MRI incompatibility, current alcohol or substance dependence disorder, or insufficient mastery of the Dutch language to perform tests in a valid way. The criteria for MRI compatibility include all the criteria for participation in the TMS study and were generally used in the larger study of apathy. Patients had to be at least 18 years of age.

Healthy controls had to be at least 18 years of age. Gender, age, and handedness were matched on a group level to the patients participating in the rTMS/NIRS session. Exclusion criteria were history or presence of psychiatric or neurological illness. All healthy controls and 12 patients were right-handed, one patient was ambidextrous.

### Ethics

All study protocols were fully approved by the medical ethical board of the University Medical Center Groningen (METC; UMCG) with the reference number METc2013.137. All procedures were carried out according to the declaration of Helsinki.

This study is part of a clinical trial registered in the Netherlands Trial Register under Clinical Trial Registry Number 3805 (https://www.trialregister.nl/trial/3659).

Each participant in the study signed written informed consent, and only those who were fully capable of making their own decision regarding participation in the study were included.

### Procedure

Upon arrival, healthy control participants filled in the remaining questionnaires (AES) before the rTMS procedure. Patients, had the questionnaires filled in on previous sessions. Subsequently, the site of the stimulation, right DLPFC, was defined as F4 electrode position (in the international 10/20 system) using the BramF3 software^60^. The software determines positions of F3 or F4 given the circumference, tragus-tragus, and inion-nasion distances. Then the F3 and F4 positions are defined by the distance from midline along circumference and distance from vertex along the *y* direction.

Next, the fNIRS cap was fitted and fNIRS optodes were placed. The participant was then laid in a comfortable dentist chair, to minimize Mayer-waves^61^. The operator agreed with the participant upon non-verbal communication methods in case of need, to avoid any talk, since the fNIRS sites were overlapping with the speech perception areas. The RTMS coil was placed above the F4 and the rTMS was delivered in a duration of ~20 min. Before the first rTMS train, a baseline fNIRS recording in a duration of a minimum 60 s was acquired.

A physician was available or on call, in case of medical intervention was required. Both patients and healthy control underwent rTMS without side-effects thus medical care was not required.

### NIRS acquisition

**Fig. 4:**
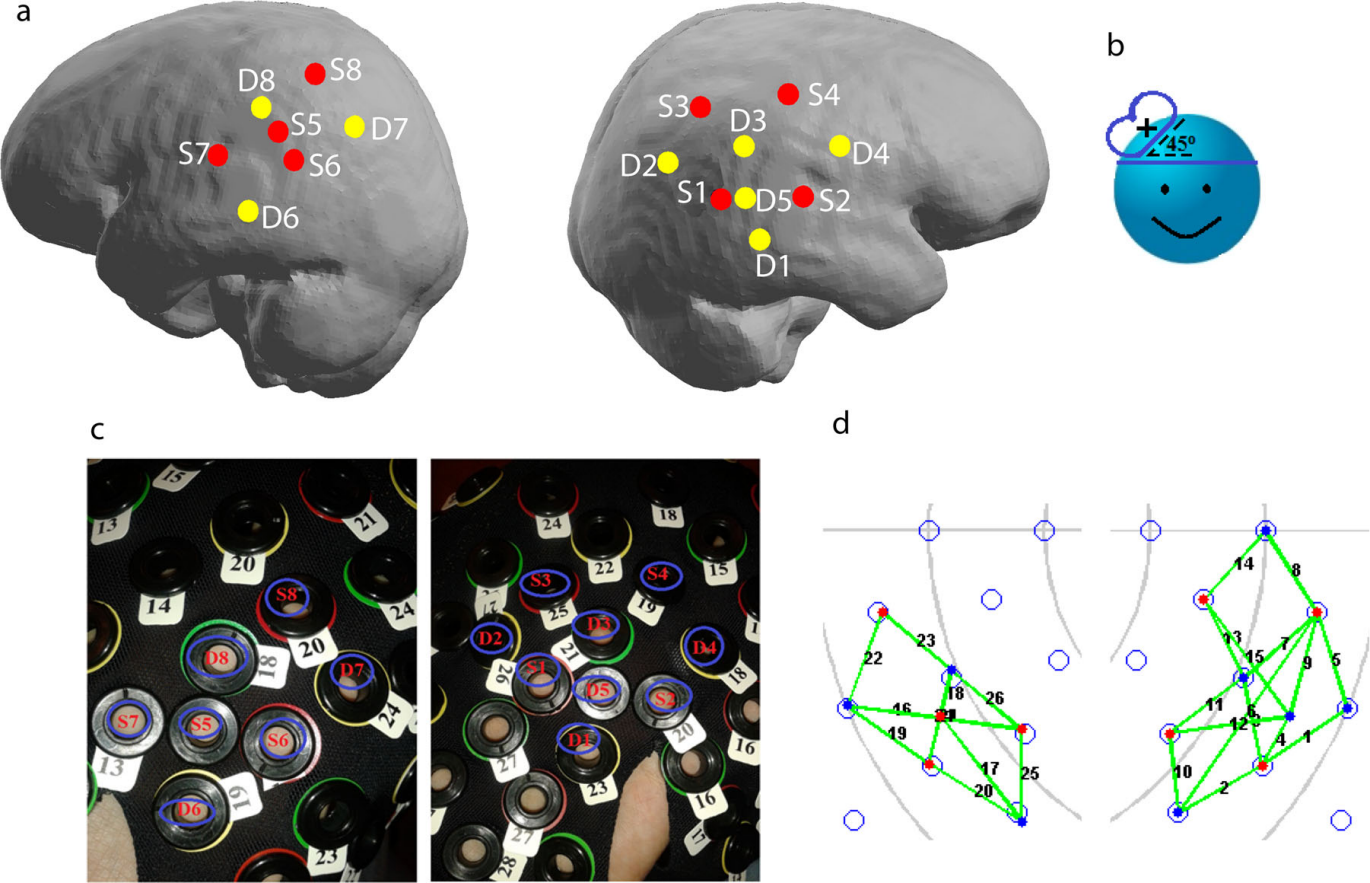
Optodes (S—sources, D—detectors) and channel placement. **a** Distribution of sources (red dots) and detectors (yellow dots) projected on the brain surface. **b** Illustration of the TMS coil orientation. **c** Placement of the sources and detectors on the cap. **d** Measured channels: right—in the ipsilateral hemisphere (channels 1–15); and left in the contralateral hemisphere (channels 16–26).

Concentration changes of oxygenated (HbO) and deoxygenated (Hb) haemoglobin were recorded by NIRScout (DYNOT, Germany) using 8 source and 8 detector optodes. The light was emitted and recorded at two wavelengths (760 and 830 nm) at a sampling frequency of 7.8125 Hz. Optodes were placed over ipsilateral and contralateral IPL (see Fig. 4). The optodes were arranged in a 3 cm mesh and one optode was placed at a shorter distance (D5 on the ipsilateral and S5 on the contralateral hemisphere). The list of channels, optodes, and distances is presented in Table 3.

**Table 3:** Channels between the sources and detectors.

Channels	Sources	Detectors	Distance [cm]
1	1	1	3
2	1	2	3
3	1	3	3
4	1	5	1.5
5	2	1	3
6	2	2	4.5
7	2	3	3
8	2	5	1.5
9	2	4	3
10	3	2	3
11	3	3	3
12	3	5	4.5
13	4	3	3
14	4	4	3
15	4	5	4.5
16	5	6	1.5
17	5	7	3
18	5	8	1.5
19	6	6	3
20	6	7	3
21	6	8	3
22	7	6	4.5
23	7	8	3
24	8	6	4.5
25	8	7	3
26	8	8	3

The left column lists the channel number. The second and the third columns from the left list the source and detectors, respectively. The channel refers to the measurements between the respective source and detector. The fourth column lists distances between sources and detectors in cm.

### rTMS

rTMS was administered by using a Magstim Rapid^2^ stimulator (Medtronic, USA) with a 70 mm Double Air figure-of-eight coil over the F4 electrode position defined as described above. rTMS consisted of 20 trains of impulses at 10 Hz for 3 seconds, and 60 seconds waiting time. Simultaneously, we measured brain activation IPL using fNIRS. The intensity was set fixed to 60% of the maximal machine output for all participants. The intensity was fixed for all participants to minimize the burden to patients who subsequently participated in a study on the treatment of apathy.

### NIRS data analysis

We estimated levels of Oxygenised haemoglobin (HbO) using NIRSLab software (NIRx Medical Technology, LLC; https://nirx.net/nirslab-1). First, data were checked for saturation (i.e., the introduction of non-number value in the absorption data due to detectors receiving too much light). No saturation was detected in our data pool. Then, data quality for each channel was examined. Channels with a coefficient of variance (CV) greater than 7.5% for either wavelength were excluded from the analysis. CV indicates the signal-to-noise ratio (SNR), with lower values implying better SNR. It is calculated from the absorption data of each measurement wavelength using the following formula: CV = 100×(Standard Deviation)/Mean (Fig. 2).

Subsequently, the fNIRS data were checked for motion artifacts through visual inspection and corrected where appropriate. If there were channels with many uncorrectable artifacts within assessment trials were excluded from the analysis. This was needed only for one subject, for which only 7 blocks were included in the analysis. To remove other physiological artifacts (e.g., slow drifts, respiration), a band-pass filter was applied to the absorption data. A low-frequency cutoff of 0.01 Hz and a high-frequency cutoff of 0.2 Hz were used with a 15% roll-off width each. Finally, the absorption data were converted into concentration data the modified Beer-Lambert Law (mBLL): $OD_\lambda = \left( {\varepsilon _{HbO_2}^\lambda \left[ {HbO_2} \right] + \varepsilon _{HbR}^\lambda \left[ {HbR} \right]} \right) \cdot DPF \cdot d + G$^62^. Here, OD denotees optical density or absorption, λ the wavelength of the light, ε denotes the extinction coefficient, the d in the formula denotes the distance between the source and the detector, while G represents the loss of light intensity due to scattering. We used the absorption spectra provided by Gratzer and associates^63^ to determine the ε of HbO and Hb for each wavelength (see Fig. 3). DPF is the differential pathlength factor and the current study used the DPFs provided by Essenpreis and colleague^64^. Eventually, the changes of concentration values were calculated.

GLM was applied using both HRF and its derivative. *t*-test was used, with FDR correction, to compare beta values of HbO levels following rTMS stimulation. HRF and derivative were defined from 0 to 32s after the onset of the rTMS train. The highest and the lowest peaks of HRF were set at 6 and 16 s, respectively, and the design matrix was filtered using Gaussian function with 4 mm FWHM.

Average timecourses were calculated per participant per time point from 5 seconds before the onset of the stimulation burst to 60 seconds after. *t*-test was used with bonferroni correction to compare time segments between the two groups.

All the statistical tests were, where relevant (such as *t*-tests), two-sided.

### Reporting summary

Further information on research design is available in the Nature Research Reporting Summary linked to this article.

## Supplementary information

Reporting Summary

## Data Availability

The data that support the findings of this study are available from the corresponding author upon reasonable request.
