# Supplementary material for: Causal connectivity from right DLPFC to IPL in schizophrenia patients: a pilot study
Source: Schizophrenia (Heidelb). 2022 Mar 7;8(1):16. doi: 10.1038/s41537-022-00216-0 (PMC8901827; doi:10.1038/s41537-022-00216-0)
Supplement: Supplementary file 1 — Reporting Summary [file 41537_2022_216_MOESM1_ESM.pdf]

nature portfolio

Corresponding author(s): Curcic-Blake Branislava

Last updated by author(s): Nov 10, 2021

Reporting Summary

Nature Portfolio wishes to improve the reproducibility of the work that we publish. This form provides structure for consistency and transparency in reporting. For further information on Nature Portfolio policies, see our Editorial Policies and the Editorial Policy Checklist.

Statistics

For all statistical analyses, confirm that the following items are present in the figure legend, table legend, main text, or Methods section.

n/a	Confirmed	
☐	☒	The exact sample size (*n*) for each experimental group/condition, given as a discrete number and unit of measurement
☒	☐	A statement on whether measurements were taken from distinct samples or whether the same sample was measured repeatedly
☐	☒	The statistical test(s) used AND whether they are one- or two-sided<br>*Only common tests should be described solely by name; describe more complex techniques in the Methods section.*
☐	☒	A description of all covariates tested
☐	☒	A description of any assumptions or corrections, such as tests of normality and adjustment for multiple comparisons
☐	☒	A full description of the statistical parameters including central tendency (e.g. means) or other basic estimates (e.g. regression coefficient) AND variation (e.g. standard deviation) or associated estimates of uncertainty (e.g. confidence intervals)
☐	☒	For null hypothesis testing, the test statistic (e.g. *F*, *t*, *r*) with confidence intervals, effect sizes, degrees of freedom and *P* value noted<br>*Give P values as exact values whenever suitable.*
☒	☐	For Bayesian analysis, information on the choice of priors and Markov chain Monte Carlo settings
☒	☐	For hierarchical and complex designs, identification of the appropriate level for tests and full reporting of outcomes
☒	☐	Estimates of effect sizes (e.g. Cohen's *d*, Pearson's *r*), indicating how they were calculated

Our web collection on statistics for biologists contains articles on many of the points above.

Software and code

Policy information about availability of computer code

Data collection	NIrsStar is a free software provided by NIRX company for recording of fNIRS data
Data analysis	NIrsLab is a free software provided by NIRS company for fNIRS data analysis. We used Matlab (a commercial program licenced to our university) to calculate averages of time courses. We used SPSS commercial program (either licenced to our university or to a university user for use at home) for statistical analyses

For manuscripts utilizing custom algorithms or software that are central to the research but not yet described in published literature, software must be made available to editors and reviewers. We strongly encourage code deposition in a community repository (e.g. GitHub). See the Nature Portfolio guidelines for submitting code & software for further information.

Data

Policy information about availability of data

All manuscripts must include a data availability statement. This statement should provide the following information, where applicable:

- Accession codes, unique identifiers, or web links for publicly available datasets
- A description of any restrictions on data availability
- For clinical datasets or third party data, please ensure that the statement adheres to our policy

The data that support the findings of this study are available from the corresponding author upon reasonable request.

Field-specific reporting

Please select the one below that is the best fit for your research. If you are not sure, read the appropriate sections before making your selection.

☒ Life sciences ☐ Behavioural & social sciences ☐ Ecological, evolutionary & environmental sciences

For a reference copy of the document with all sections, see nature.com/documents/nr-reporting-summary-flat.pdf

Life sciences study design

All studies must disclose on these points even when the disclosure is negative.

Sample size	27
Data exclusions	n/a
Replication	This is a pilot study
Randomization	The diagnosis was made by clinician and confirmed using MINI interview.
Blinding	N/a

Reporting for specific materials, systems and methods

We require information from authors about some types of materials, experimental systems and methods used in many studies. Here, indicate whether each material, system or method listed is relevant to your study. If you are not sure if a list item applies to your research, read the appropriate section before selecting a response.

Materials & experimental systems

n/a	Involved in the study	
☒	☐	Antibodies
☒	☐	Eukaryotic cell lines
☒	☐	Palaeontology and archaeology
☒	☐	Animals and other organisms
☐	☒	Human research participants
☐	☒	Clinical data
☒	☐	Dual use research of concern

Methods

n/a	Involved in the study	
☒	☐	ChIP-seq
☒	☐	Flow cytometry
☒	☐	MRI-based neuroimaging

Human research participants

Policy information about studies involving human research participants

Population characteristics	Healthy controls: n=14, age 36 (13.7), males/female 8/6, education according to Dutch scale by Verhage 6.3 (0.6)<br><br>patients with schizophrenia: n=13, age 39.2 (11.4), males/female 10/3, education according to Dutch scale by Verhage 5.5 (0.9)
Recruitment	Patients were recruited from several psychiatric hospitals from different parts of the Netherlands, whom have made a commitment to participate in this study: GGZ Drenthe, GGZ Friesland, Lentis Center for Mental Health Care (Zuidlaren, Winschoten and Groningen), the department of Psychiatry at the UMCG, and the Department of Psychiatry UMC Utrecht. Furthermore ealthy controls, they were recruited in Groningen by advertisement and by word of mouth.<br><br>Only patients who decided to participate in this specific part of the trial were recruited. That means that probably patients with lower levels of apathy participated for this part of the trial. Nevertheless, clearly patients had higher levels of apathy than healthy controls, properly representing the patient group.
Ethics oversight	University Medical Center Groningen (METC; UMCG) with the reference number METc2013.137

Note that full information on the approval of the study protocol must also be provided in the manuscript.

Clinical data

Policy information about clinical studies

All manuscripts should comply with the ICMJE guidelines for publication of clinical research and a completed CONSORT checklist must be included with all submissions.

Clinical trial registration	This study is part of clinical trial registered in the Netherlands Trial Register under Clinical Trial Registry Number 3805 (https://www.trialregister.nl/trial/3659).
Study protocol	The study protocol was approved by METC of the UMCG reference number METc2013.137 and is available upon request from researcher.<br>Otherwise details are also found in https://www.trialregister.nl/trial/3659
Data collection	Data was collected in the NIRS lab of the Cognitive neuroscience center (CNC) of the UMCG. Planned start date of this study was Start date<br>2012-09-01<br><br>but the data reported here were collected between<br>2014-08-20 and 2018 -07-26
Outcomes	Change in the oxygenated Hb levels in the inferior parietal lobe as a consequence of stimulation to the dorsolateral prefrontal cortex with 10Hz rTMS
